# Real-time cardiac power output index predicts imminent need for extracorporeal membrane oxygenation after heart transplantation^1^

**DOI:** 10.1016/j.jhlto.2026.100597

**Published:** 2026-05-20

**Authors:** Awab Ahmad, Aniket S. Rali, Chen Chia Wang, Mark Petrovic, Aaron M. Williams, John Trahanas, Swaroop Bommareddi, Tarek Absi, Eric Quintana, Kevin McGann, Stephen Devries, Joshua Lowman, Hasan Siddiqi, Marshall Brinkley, Stacy Tsai, Jonathan N. Menachem, Dawn Pedrotty, Suzanne Sacks, Sandip Zalawadiya, Rei Ukita, Matthew Bacchetta, Kelly Schlendorf, Ashish S. Shah, JoAnn Lindenfeld, Brian Lima

**Affiliations:** a Vanderbilt University Medical Center, Department of Cardiac Surgery, Nashville, Tennessee; b Vanderbilt University School of Medicine, Department of Cardiology, Nashville, Tennessee; c Vanderbilt University School of Medicine, Nashville, Tennessee; d Department of Biomedical Engineering, Vanderbilt University, Nashville, Tennessee

**Keywords:** Cardiac power output, Primary graft dysfunction, ECMO, Heart transplant

## Abstract

**Background:**

Primary graft dysfunction (PGD) remains the leading cause of early morbidity and mortality after heart transplantation. Despite consensus definitions, clinicians still rely on subjective, indirect assessments of graft performance to determine when to initiate ECMO after transplant concludes risking delayed support initiation. No real-time, physiology-based marker currently guides early PGD recognition or standardizes ECMO decision-making across centers.

**Objective:**

To evaluate the utility of the cardiac power output index adjusted for vasoactive support (CPOI-VIS) as an early, dynamic, and clinically actionable physiologic marker of PGD, capable of predicting imminent ECMO requirement and short-term outcomes after heart transplantation.

**Methods:**

All adult heart transplant recipients at a single center (January 2020 to June 2025 [n = 547]) were retrospectively analyzed. Multiorgan recipients, congenital heart disease, and patients leaving the operating room on ECMO were excluded. CPOI-VIS was calculated hourly for the first 72 postoperative hours and censored at ECMO initiation. Dynamic 6-hour risk modeling, rolling ROC analyses, and unsupervised functional trajectory phenotyping was used to characterize CPOI-VIS behavior and its association with ECMO within 72 hours. The primary endpoint was ECMO initiation; secondary endpoints included 90-day mortality, renal replacement therapy, and ICU length of stay.

**Results:**

Sixteen patients (2.9%) required ECMO within 72 hours. CPOI-VIS values diverged immediately between ECMO and non-ECMO groups (*p* < 0.001). For every 1-unit decrease in CPOI-VIS, the associated hazard of ECMO in the next 6 hours more than doubled (HR 2.23; 95% CI 1.66-2.99; C-index 0.96). Across 0 to 72 hours, discrimination remained consistently high (median AUC 0.98; IQR 0.96-0.99) with a stable predictive threshold (median 4.8; IQR 3.9-5.7). Among those crossing CPOI-VIS < 4.8 W/m^2^, early ECMO (<24 hours) was associated with markedly lower 90-day mortality compared with later initiation (0% vs 40%), with a >93% posterior probability of benefit. A “low CPOI-VIS” trajectory was strongly associated with ECMO, renal failure, prolonged ICU stay, and increased 90-day mortality.

**Conclusions:**

CPOI-VIS is a robust, physiology-based, real-time marker of early graft dysfunction after heart transplantation that reliably identifies patients at high risk for imminent hemodynamic collapse and may guide earlier ECMO initiation.

## Graphical abstract

**Figure:**
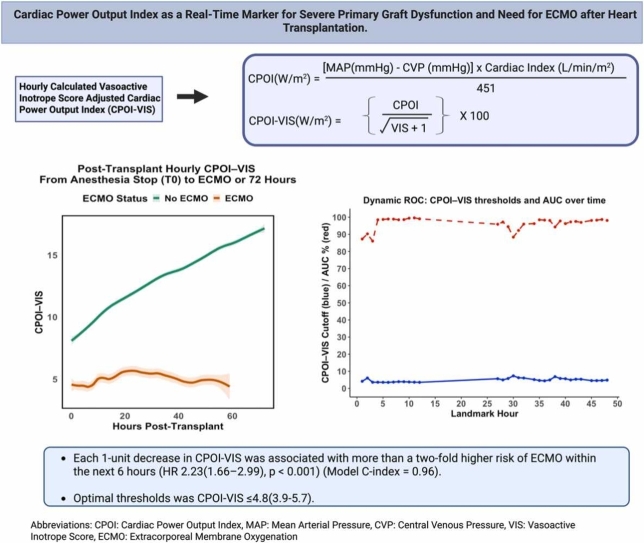

Primary graft dysfunction (PGD) remains one of the most feared complications following heart transplantation,^1^ occurring in approximately 3% to 30% of recipients and contributing substantially to early postoperative morbidity and mortality with several series reporting early mortality exceeding 50% in severe cases.^2^, ^3^ To improve consistency in diagnosis and reporting, the International Society for Heart and Lung Transplantation (ISHLT) established consensus criteria in 2014.^3^ Severe PGD is defined in part by the need for venoarterial extracorporeal membrane oxygenation (ECMO) within 24 hours of transplant, whereas moderate PGD is characterized by ancillary hemodynamic indices such as low cardiac index, high vasoactive inotrope score (VIS), or the need for intra-aortic balloon pump (IABP) support.^3^

Although these consensus criteria have been essential for standardizing PGD definitions,^4^, ^5^ they also expose a fundamental dilemma. A definition based largely on treatment, particularly ECMO initiation introduces inherent circularity, institutional practice bias, and leads to definition bias or reverse causality.^6^ Clinicians cannot reliably identify severe PGD before it becomes clinically catastrophic if its defining feature is the initiation of rescue therapy. This framework risks delayed treatment in patients who might benefit from earlier support and makes it difficult to compare practice patterns across centers.

In the operating room, transplant surgeons rely on direct visual assessment of allograft performance, intraoperative hemodynamics, and the ability to wean from cardiopulmonary bypass. When overt graft failure is evident, such as failure to separate from bypass or severe contractile dysfunction, ECMO is often instituted before leaving the operating room. However, more commonly, the transplant concludes with the heart marginally stable. Once the chest is closed and the patient transitions to the intensive care unit (ICU), detection of deteriorating graft function becomes more complex. Clinicians must rely on their judgment that might be based on a constellation of indirect markers such as cardiac index, blood pressure, lactate, VIS, echocardiographic findings, and signs of end-organ perfusion, each reflecting only one dimension of graft physiology.^1^, ^3^ The timing of ECMO initiation therefore varies widely by clinician experience, perceived reversibility, and institutional culture rather than by a standardized physiologic metric.

This subjectivity leaves an important gap: there is no universally accepted, quantitative, dynamic marker of post-transplant cardiac performance that reliably identifies early graft dysfunction and guides timely ECMO decision-making.^1^, ^7^ Timing is critical when mechanical circulatory support is needed. Delayed ECMO can allow fulminant shock and multi-organ injury to develop, whereas timely initiation may stabilize the patient and provide the allograft an opportunity to recover.^7^, ^8^, ^9^, ^10^

In the broader context of cardiogenic shock and advanced heart failure, cardiac power output (CPO) and its indexed form, cardiac power output index (CPOI), have emerged as powerful, integrative markers of global cardiac performance.^11^, ^12^, ^13^, ^14^, ^15^ CPOI incorporates flow (cardiac output) and perfusion pressure (mean arterial pressure) into a single metric of mechanical work per body surface area, reflecting the heart’s ability to deliver hydraulic power to the circulation; its fundamental function.^11^, ^15^ Fincke et al. demonstrated that cardiac power was the strongest hemodynamic correlate of mortality in the SHOCK trial, outperforming cardiac index, filling pressures, and systemic vascular resistance.^11^ Subsequent studies in heart failure, coronary artery disease, and transcatheter aortic valve implantation have reinforced the prognostic superiority of cardiac power over traditional parameters.^12^, ^13^, ^14^ In the transplant setting, early CPOI has been associated with severe PGD and worse outcomes.^16^

Importantly, CPOI alone does not account for the level of pharmacologic support required to sustain hemodynamics. Adjusting CPOI for VIS yields a vasoactive-integrated measure, CPOI-VIS, that quantifies “effective cardiac power” per unit of inotropic and vasopressor support. This ratio captures both the heart’s intrinsic mechanical performance and its responsiveness to pharmacologic augmentation, thereby offering a standardized, physiologically coherent, and interpretable index of myocardial efficiency.^16^

We hypothesized that CPOI-VIS could serve as a real-time, dynamic marker of early graft dysfunction after heart transplantation, capable of identifying patients at increased risk of imminent ECMO requirement and highlighting physiologic states of concern. Leveraging a large, contemporary transplant cohort with granular hourly hemodynamics, we sought to evaluate how early CPOI-VIS discriminates patients who require ECMO; explore whether a consistent range of values associated with increased risk can be identified; assess whether timing of ECMO relative to this range influences survival; and define phenotypic patterns of early graft performance.

## Methods

### Population

**Figure 1:**
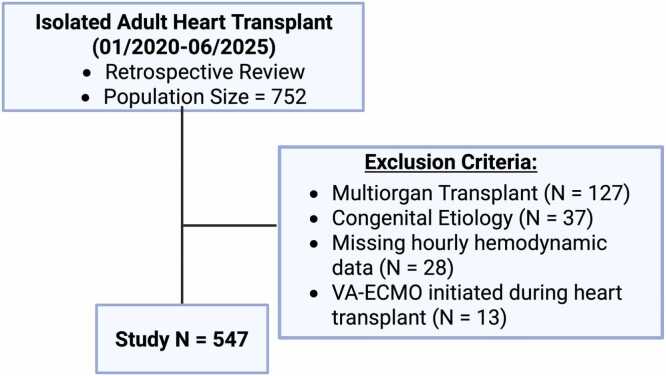
Population selection flowchart

All adult heart transplant recipients from January 2020 to June 2025 were retrospectively analyzed. Multiorgan transplant and congenital etiology of heart failure cases were excluded. Patients who came out of surgery on ECMO were also excluded. (Figure 1).

### Exposure

Cardiac power output index (CPOI) and vasoactive-inotropic score (VIS) were calculated hourly from anesthesia stop time (T0) to 72 hours post-transplant using hemodynamic parameters continuously tracked during postoperative ICU care, as described in the literature.^11^, ^16^

CPOI = ([MAP (mmHg) – CVP (mmHg)] X CI (L/min/m^2^)) / 451

Where MAP = Mean Arterial Pressure, CVP = Central Venous Pressure and CI = Cardiac Index.

A VIS adjusted CPOI metric (CPOI-VIS) was calculated as described by Lim et al.^16^:

CPOI-VIS = (CPOI/ √(VIS+1)) X 100

Where VIS = dobutamine + dopamine + 100 × (noradrenaline + adrenaline) +15 × milrinone.^3^ All drug dosages were measured as μg/kg/min.

Hemodynamic measurements were censored at the time of ECMO initiation.

### Outcomes

The primary outcome was initiation of ECMO within 72 hours after transplantation. Secondary outcomes included 30-day, 90-day, and 1-year survival; need for renal replacement therapy (RRT); and ICU length of stay.

### Objectives and prespecified performance criteria

Post-transplant CPOI-VIS was evaluated as a hemodynamic marker of early graft dysfunction. A priori, we prespecified that an informative marker should demonstrate a strong association with graft dysfunction, operationalized as ECMO requirement; accurately predict short-term risk over time with a target AUC >0.80; yield a generalizable threshold that could guide timely ECMO initiation and be associated with lower early mortality; and show consistent associations with other adverse outcomes, including RRT, ICU length of stay, and 90-day mortality.

The study was approved by the Vanderbilt University Medical Center Institutional Review Board (# 241909), with a waiver of informed consent due to the retrospective nature of the analysis.

### Statistical analysis

1. Trajectory Description and Group Comparisons
  Patients were grouped by whether they remained ECMO-free through 72 hours post-transplant or required ECMO support. CPOI-VIS trajectories were summarized as medians with standard errors at prespecified postoperative hours (3, 6, 12, 18, 24, 48, 60, and 72). Between-group comparisons at each hour were performed using Wilcoxon rank-sum tests, with Benjamini–Hochberg false-discovery-rate (FDR) correction applied across time points. Patients were censored from further comparisons following ECMO initiation. Parallel analyses for CPOI and VIS alone are presented in Supplemental Section 1.
2. Dynamic Time-Updating Risk Models for ECMO
  To quantify the evolving short-term risk of ECMO, we employed complementary time-to-event frameworks. Dynamic Cox “moving-window” models estimated the hazard of ECMO within the subsequent 6 hours (t, t+6] based on the current CPOI-VIS value at hour t, using robust standard errors clustered by patient to account for repeated risk intervals.
  We also fit joint longitudinal–time-to-event models that linked linear mixed-effects models of the CPOI-VIS trajectory (with random intercept and slope and flexible fixed effects for time) to a Cox proportional hazards model for time to ECMO. Both current value and short-term slope of CPOI-VIS were evaluated to determine whether absolute level or rate of decline best predicted risk. Hazard ratios, Harrell’s C-index, posterior means, and 95% credible intervals are reported (Supplemental Section 2 shows parallel CPOI and VIS-based analysis).
3. Rolling ROC Analysis and Threshold Identification
  To assess discrimination and derive actionable physiologic thresholds, rolling ROC analyses were performed at each postoperative hour from 0 to 72. At each hour, we calculated the area under the curve (AUC) for predicting ECMO within the next 6 hours and identified the optimal cutoff using the Youden index. We summarized the median (IQR) of hourly thresholds and AUCs to evaluate temporal stability and generalizability. The same procedure was applied to CPOI and VIS individually (Supplemental Section 3). Because hourly landmark windows can contain few ECMO events, we also performed a pooled discrimination analysis across all landmark risk sets to provide a more stable summary of overall predictive performance. Patient–hour observations were pooled across postoperative hours 1 to 48, and AUC was calculated for predicting ECMO within the subsequent 6 hours. Internal validation was performed using patient-level bootstrap resampling with out-of-bag evaluation to estimate optimism-corrected AUC and assess cutoff stability, preserving within-patient correlation across repeated measurements.
4. Threshold-Anchored Early vs Late ECMO Sensitivity Analysis
  For clinical interpretability, we defined “threshold crossing” using the landmark-derived median cutoff and classified ECMO as early if initiated within 24 hours of first crossing and late otherwise. ECMO initiation was considered “early” if performed within 24 hours of threshold crossing and “late” otherwise. Among threshold crossers, 90-day mortality was analyzed using a Bayesian small-sample framework with Jeffreys’ prior for binomial proportions. Posterior risks, 95% credible intervals, and the posterior probability of mortality reduction with early ECMO were calculated. Analogous analyses for CPOI and VIS thresholds are provided in Supplemental Section 4.
5. Unsupervised Trajectory Phenotyping and Predictors of Low CPOI-VIS
  To identify distinct patterns of early graft performance, we performed functional principal component analysis (FPCA) of hourly CPOI-VIS, followed by k-means clustering (diagnostics in Supplemental Section 5). Baseline donor and recipient characteristics were summarized using medians (IQR) or counts (%) and compared across phenotypes with Kruskal–Wallis and Fisher’s exact tests.
  Pre-transplant predictors of the low CPOI-VIS phenotype were evaluated using L1-regularized logistic regression (LASSO). Candidate variables were selected a priori from clinical relevance and data availability; the penalty parameter (λ) was chosen by 10-fold cross-validation to minimize binomial deviance.
6. Independent Effects of Low CPOI-VIS Phenotype on Outcomes
  To estimate independent effects of the low CPOI-VIS phenotype on key outcomes, we applied inverse-probability-of-treatment weighting (IPTW) using generalized boosting model to balance prespecified covariates across phenotypes (balance assessments in Supplemental Section 5). Weighted Firth-penalized logistic regression was used for binary outcomes (ECMO within 72 hours, RRT, 90-day mortality), and weighted quantile regression (τ = 0.50) was used for continuous outcomes such as ICU length of stay.
7. Model Assumptions, Validation, and Multiple Testing

Cox proportional hazards assumptions were evaluated using time-interaction probes and Schoenfeld-type diagnostics adapted to the moving-window design. Convergence and mixing of joint longitudinal–time-to-event models were assessed via R̂ statistics and visual inspection of posterior samples. Clustering stability was evaluated using internal resampling. Multiple hypothesis testing across time points was controlled using Benjamini–Hochberg FDR. Two-sided α = 0.05 was used for statistical significance unless otherwise specified.

All analyses were performed using R version 4.4.1.

## Results

### Study cohort

Among 547 heart transplant patients (median age 59 years (IQR: 49-65); 140 (25.6%) female), 16 (2.9%) required ECMO within 72 hours.

### Early divergence of CPO-VIS between groups

**Figure 2:**
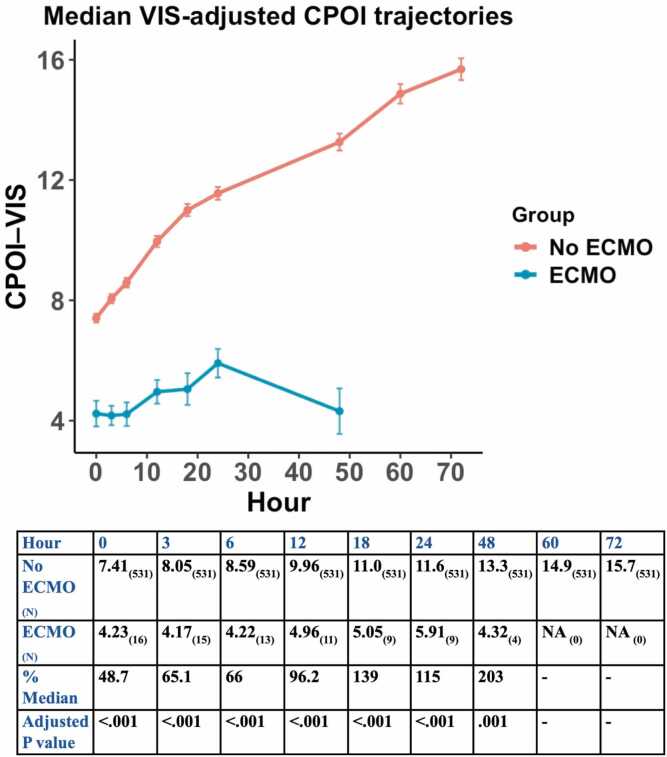
Median difference in Vasopressor adjusted Cardiac Power Output Index (CPOI-VIS) between ECMO and non-ECMO cases at 0, 3 h, 6 h, 12 h, 18 h, 24 h, 48 h, 60 h, and 72 h, respectively and *p* value adjusted for multiple comparisons. (CPOI-VIS values censored at ECMO initiation)

Across the first 72 postoperative hours, median CPOI-VIS values were consistently lower among patients who required ECMO than those who did not (Figure 2). The difference was evident as early as hour 0 post-transplant and widened progressively through 72 hours (all FDR-adjusted *p* < 0.05). For example, at 12 hours, the median CPOI-VIS was 4.2 (IQR 3.4-5.2) in the ECMO group vs 8.6 (IQR 6.5-11.3) in the non-ECMO group (*p* < 0.001). Parallel findings for unadjusted CPOI and VIS are included in Supplemental Table S1.

### Dynamic prediction of ECMO risk

In the dynamic Cox time-varying model, lower CPOI-VIS values were strongly and consistently associated with a higher short-term hazard of ECMO initiation. For every 1-unit decrease in CPOI-VIS, the associated instantaneous hazard of ECMO within the subsequent 6 hours increased more than 2-fold (HR 2.23, 95% CI 1.66-2.99; *p* < 0.001, C-index 0.96). The association remained stable when examined across sequential hourly intervals. In the joint longitudinal–time-to-event framework linking the CPOI-VIS trajectory to time to ECMO, the current value of CPOI-VIS emerged as a strong independent determinant of risk. Each 1-unit lower current CPOI-VIS value was associated with an ∼1.85-fold higher instantaneous hazard of ECMO (posterior mean HR 1.85; 95% credible interval 1.33-2.63). The short-term slope of CPOI-VIS also appeared directionally consistent (posterior mean coefficient −24.95; 95% CrI −38.32 to −7.78), suggesting that rapid decline may further amplify risk prediction; however, the MCMC (Markov Chai Monte Carlo) convergence for the slope term was suboptimal (R̂ = 1.76), so these results are interpreted cautiously. In contrast, convergence for the current-value term was excellent (R̂ = 1.01).

### Stable and actionable threshold for imminent ECMO

**Figure 3:**
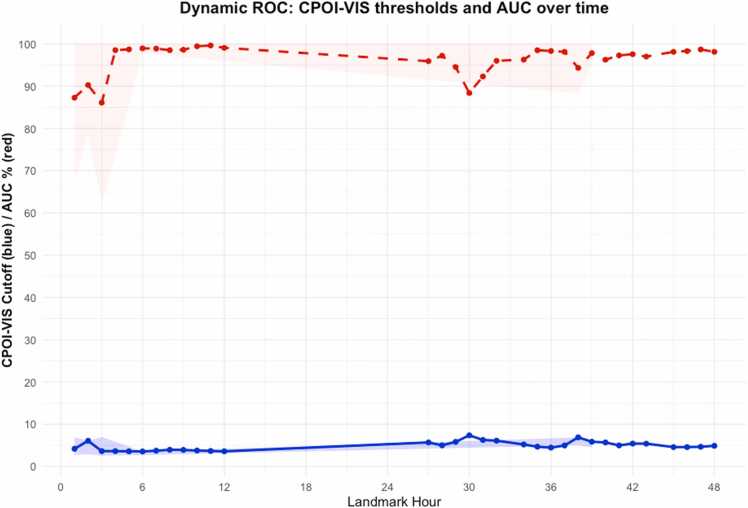
Time-updating discrimination and threshold estimates for CPOI-VIS. Dynamic receiver operating characteristic (ROC) analysis demonstrating time-varying discrimination of CPOI-VIS for predicting ECMO within the subsequent 6 hours across postoperative landmark time points (hours 1-48). The dashed red line represents the area under the curve (AUC), and the blue line represents the corresponding Youden index-derived threshold at each time point. Shaded regions indicate 95% confidence intervals for AUC (red) and threshold estimates (blue), displayed for landmark time points with ≥3 events. Given the low number of ECMO events at individual time points, these hour-specific estimates should be interpreted with caution

Across hourly postoperative evaluations (0-72 hours), CPOI-VIS demonstrated stable, high discrimination for predicting ECMO within the subsequent 6 hours, with a median AUC of 0.98 (IQR 0.96-0.99). The corresponding optimal threshold was highly consistent over time (median 4.8, IQR 3.9-5.7). (Figure 3) Parallel analyses of the component markers yielded similarly strong performance. (Supplemental Section 3). There were 102 patients with CPOI-VIS ≤ 4.8 during first 4 postoperative hours, 35 (34%) demonstrated recovery with CPOI-VIS rising above threshold after 4 hours, whereas 67 (66%) had continued low values beyond 4 hours. ECMO requirement differed across these patterns: 2 of 35 patients (5.7%) with transient early low and 10 of 67 (14.9%) with persistent early low required ECMO. Overall, 90 of 102 patients (88.2%) remained ECMO-free.

### Early ECMO Relative to Threshold is Associated with Lower Risk of 90-day Mortality

In the sensitivity analysis anchored to the landmark-derived median threshold (CPOI-VIS ≤4.8 W/m²), among ECMO patients who first crossed the threshold, early ECMO (≤ 24 hours) had 0/5 deaths vs 3/9 deaths with later ECMO (> 24 hours). Under a Jeffreys prior, the posterior 90-day mortality was ∼8% for early vs ∼35% for late, with a 93.6% posterior probability that early ECMO is associated with reduced mortality among recipients with graft dysfunction. (Supplemental Section 4).

### Crossing the threshold without ECMO was also associated with 90-day mortality

To assess whether the CPOI-VIS threshold carries prognostic information independent of ECMO use, we examined patients who did not receive ECMO and stratified them by whether they crossed the threshold CPOI-VIS ≤ 4.8 W/m^2^ in the 72-hour postoperative period. Mortality at 90 days was 4.7% (7/150) among those who crossed vs 1.6% (6/381) among those who did not, (OR 3.02, 95% CI: 1.03-9.11, *p* = 0.04).

In the Pooled analysis across all landmark risk sets (25,893 patient–hour observations; 63 six-hour ECMO events), discrimination remained high (AUC 0.951). Patient-level bootstrap out-of-bag validation demonstrated minimal optimism (0.0016), yielding an optimism-corrected AUC of 0.950 (OOB median 0.95; 95% CI 0.91-0.99). The pooled Youden cutoff was stable under bootstrap resampling (median 6.28 W/m²; IQR 6.28-6.96). The slightly higher bootstrap-derived cutoff suggests modest variability in threshold estimation, supporting interpretation of CPOI-VIS thresholds as descriptive rather than fixed decision boundaries.

### Machine-learning phenotypes and independent effects on outcomes

**Figure 4:**
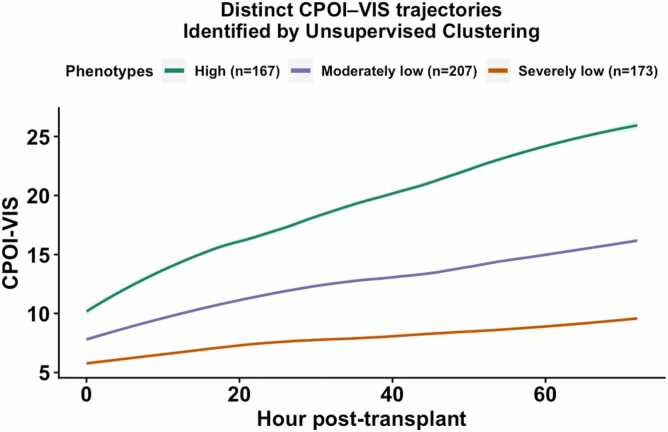
Unsupervised machine learning identified distinct vasopressor adjusted cardiac power output index (CPOI-VIS) trajectory phenotypes within the first 72 h post-transplant

**Figure 5:**
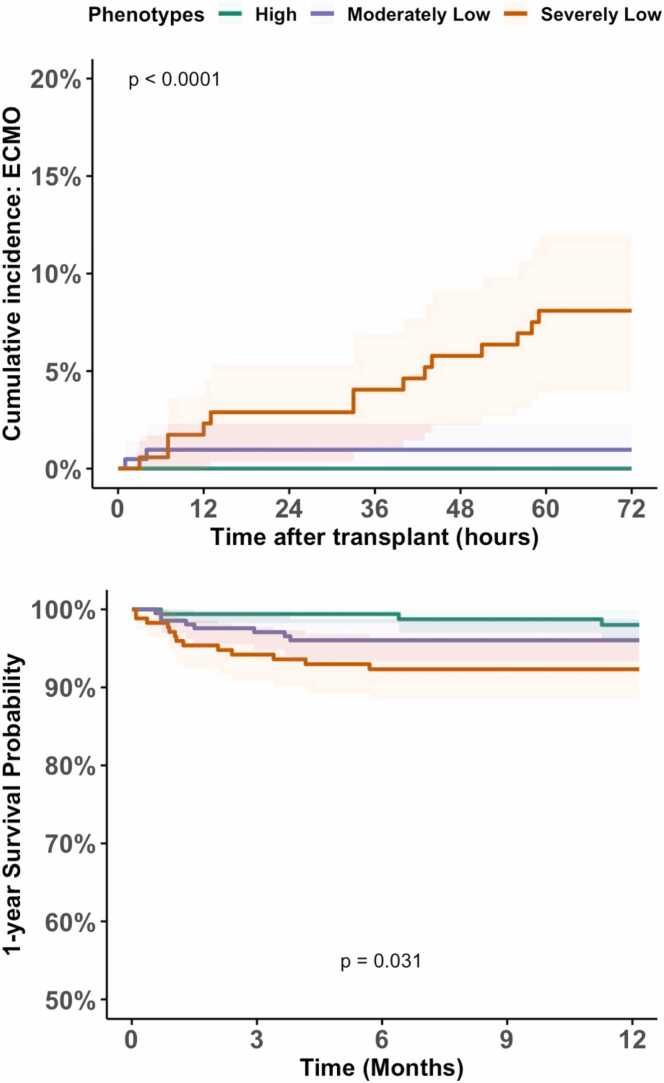
Time-to-event analyses using Cox Hazard Models: first figure showing increased cumulative incidence of need for ECMO within 72 hours post-transplant in the severely low CPOI-VIS phenotype. Second figure showing worst 1-year survival in the severely low CPOI-VIS phenotype

Unsupervised machine learning uncovered 3 CPOI-VIS phenotypes with distinct levels: High (median 18.2 W/m^2^ [IQR 16.3-20.8]), Moderate (median 12.3 W/m^2^ [11.2-13.5]), and Low (median 7.7 W/m^2^ [6.3-8.9]; *p* < 0.001) (Figure 4). Baseline characteristics are summarized in Table 1, Table 2. The Low phenotype showed a higher cumulative incidence of ECMO and worse 1-year survival than the other groups (Figure 5).

**Table 1:** Baseline Donor and Recipient Differences Among Distinct VIS Adjusted Cardiac Power Output Index Phenotypes

	**Total N = 547**	**Phenotype 1 N = 167 (30.5%)**	**Phenotype 2 N = 207(37.8%)**	**Phenotype 3 N = 173(31.6%)**	***p* Value**
**Donor Data**					
Sex, female	164(30%)	51(30.5%)	62(30%)	51(29.5%)	0.99
Age, years	32(24-41)	31(24-40)	33(25-41)	32(24-41)	0.69
Procurement Type					0.89
DBD	335(61.2%)	104(62.3%)	124(59.9%)	107(61.8%)	
DCD	212(38.8%)	63(37.7%)	83(40.1%)	66(38.2%)	
Preservation Type					0.49
Static Storage	479(87.6%)	150(89.8%)	181(87.4%)	148(85.6%)	
Machine Perfusion	68(12.4%)	17(10.2%)	26(12.6%)	25(14.4%)	
Machine Perfusion Type					0.42
Normothermic	45(8.2%)	11(6.6%)	20(9.7%)	14(8.1%)	
Hypothermic	23(4.2%)	6 (3.6%)	6(2.9%)	11 (6.4%)	
Static Storage Type					0.56
Ice	271(49.5%)	90(53.9%)	97(46.9%)	84(48.6%)	
10°C	207(37.8%)	60(35.9%)	84(40.6%)	63(36.4%)	
4-8°C	1(0.2%)	0(0%)	0(0%)	1(0.6%)	
Donor Distance, nautical miles	317(158-456)	317(152-456)	316(149-450)	315(162-511)	0.75
Total Ischemic Time, mins	224(189-265)	216(190-256)	227(183-264)	230(193-280)	0.06
PHM ratio	0.94(0.83-1.09)	0.99(0.88-1.15)	0.93(0.82-1.08)	0.90(0.81-1.02)	**<.001**
**Recipient Data**					
Sex, female	140(25.6%)	39(23.4%)	58(28%)	43(24.9%)	0.58
Age, years	59(49-65)	60(50-65)	58(46-64)	58.5(51-65)	0.18
BMI, kg/m2	29.5(25.9-33.3)	28.2(24.8-31.2)	31.1(26.3-34.4)	30(26.1-33.6)	**<.001**
Ischemic cardiomyopathy	162(29.6%)	45(27%)	58(28%)	59(34.1%)	0.29
Diabetes	218(39.8%)	66(39.5%)	75(36.2%)	77(44.5%)	0.25
Hypertension	370(67.6%)	112(67.1%)	146(70.5%)	112(64.7%)	0.47
Prior Sternotomy	286(52.3%)	59(35.3%)	113(54.6%)	114(65.9%)	**<.001**
Hospitalized		127(48.5%)	84(40.8%)	24(30.4%)	**0.01**
Pre-transplant MCS					
IABP	91(16.6%)	36(21.6%)	35(16.9%)	20(11.6%)	**0.046**
Impella	47(8.6%)	17(10.2%)	17(8.2%)	13(7.5%)	0.68
LVAD	163(29.8%)	25(15%)	70(33.8%)	68(39.3%)	**<.001**
RVAD	11(2.01%)	3(1.8%)	4 (1.9%)	4(2.3%)	>.99
Pre-transplant ECMO	16(2.9%)	4(2.4%)	11(5.3%)	1(0.6%)	**0.02**

DBD, Donation after brain death; DCD, Donation after circulatory death; IABP, Intra-aortic Balloon Pump; LVAD, Left Ventricular Assist Device; MCS, Mechanical Circulatory Support; PHM, Predicted Heart Mass; RVAD, Right Ventricular Assist Device

**Table 2:** Comparison of Postoperative Data Among the Three VIS Adjusted Cardiac Power Output Index Phenotypes

	**Total N = 547**	**Phenotype 1 N = 167 (30.5%)**	**Phenotype 2 N = 207(37.8%)**	**Phenotype 3 N = 173(31.6%)**	***p* Value**
**Postoperative Data**					
**VIS adjusted CPOI**					
Mean	12.2 (9.37-16.2)	18.1(16.4-20.9)	12.3(11.3-13.7)	7.8(6.5-9.2)	**<.001**
Median	12.2(9.2-16)	18.2(16.3-20.8)	12.3(11.2-13.5)	7.7(6.3-8.9)	**<.001**
Nadir (0-12 h)	6.3(4.9-8.4)	9.10(6.8-10.8)	6.4(5.3-8.02)	4.7(3.8-5.6)	**<.001**
NTV	6.8(5.3-8.6)	5.4(4.2-6.9)	6.7(5.6-8.6)	8.13(6.8-9.5)	**0.02**
Slope (0-6 h)	+0.13(−0.06, +0.39)	+0.26(−0.02, +0.56)	+0.13(−0.06, +0.39)	+0.05(−0.08,+0.21)	**<.001**
Time below, hr					**<.001**
5	0(0-1.6)	0(0-0)	0(0-0)	3.1(0-12)	
10	16.9(4.5-7.6)	1.96(0-7.6)	16.3(9.0-25.1)	58.7(44-70.5)	
Area below					**<.001**
5	0(0-0.70)	0(0-0)	0(0-0)	1.55(0-8.26)	
10	31.3(5.15-95.2)	1.55(0-14.1)	27.8(11.1-52.6)	160(94.2-225)	
**Unadjusted CPOI**					
Mean	0.42(0.36-0.48)	0.51(0.47-0.57)	0.42(0.39-0.46)	0.35(0.31-0.37)	**<.001**
Median	0.42(0.36-0.49)	0.51(0.47-0.56)	0.42(0.39-0.46)	0.34(0.30-0.37)	**<.001**
Nadir (0-12 h)	0.29(0.24-0.36)	0.36(0.31-0.41)	0.30(0.25-0.35)	0.24(0.21-0.28)	**<.001**
NTV	8.7(7.2-10.1)	7.9(6.6-9.5)	8.8(7.4-10.1)	9.1(7.5-10.6)	**<.001**
Slope (0-6 h)	+0.004(−0.005, +0.01)	+0.006(−0.004, +0.01)	+0.005(−0.004, +0.014)	+0.002(−0.005, +0.008)	**0.02**
Time below 0.3, hours	3.17(0-10.7)	0(0-0.7)	2.45(0-6.9)	14.9(8.1-31.2)	**<.001**
Area below 0.3	0.1(0-0.44)	0(0-0.04)	0.06(0.0-0.24)	0.64(0.26-1.41)	**<.001**
**VIS**					
Mean	12.2(8.81-17.0)	7.86(6.04-9.37)	12.2(10.4-14.7)	20.1(15.7-25.9)	**<.001**
Median	11.4(7.46-16.0)	6.41(5-8)	11.5(8.9-13.5)	19.7(14.6-25.5)	**<.001**
Max (0-12 h)	24.1(17.9-31.5)	19.4(13.3-24.6)	23.5(18-30.3)	31(24-37.4)	**<.001**
Slope (0-6 h)	−0.23(−0.9, +0.11)	−0.34(−0.96, +6.1x10^−17^)	−0.21(−0.78, +0.009)	−0.06(−0.77, +0.37)	**0.01**
Time above, h					**<.001**
15	15.6(3.46-39.3)	3.90(0-10.4)	14.9(4.35-27.5)	55.8(29.8-72)	
20	4.7(0-18.9)	0(0-3.4)	4.1(0-13.9)	30.9(10.5-54.6)	
Area above,					**<.001**
15	64.7(8.7-250)	8.44(0-49.4)	59.9(10.6-168)	362(142-651)	
20	15.3(0-98.3)	0(0-11.3)	10.9(0-62.9)	138(0-98.3)	
CPB length, minutes	138(105-169)	128(104-151)	142(107-168)	150(112-183)	**<.001**
Severe Post-Bypass RV dysfunction	5(0.53)	1(0.6%)	1(0.5%)	3(1.7%)	0.53
Post-Bypass LVEF < 55%	51(9.3%)	11(6.6%)	15(7.3%)	25(14.4%)	**0.02**
ECMO within 72 h	16(2.9%)	0(0%)	2(0.97%)	14(8.1%)	**<.001**
RRT	111(20.3%)	12(7.2%)	36(17.4%)	63(36.4%)	**<.001**
LVEF POD7 < 55%	71(13%)	26(15.6%)	21(10.1%)	24(13.9%)	0.51
Ventilation time, hours	12(8.5-22)	9.6(7.3-13.1)	12.7(9.1-25.7)	17.1(10.6-39.4)	**<.001**
ICU LOS	7(5-11)	5(4-7)	7(5-9)	11(7-20)	**<.001**
Mortality 30 day	9(1.65%)	1(0.6%)	3(1.5%)	5(2.9%)	0.28
Mortality 90 day	17(3.1%)	1(0.6%)	6(2.9%)	10(5.8%)	**0.02**
Mortality 1 yr	24(4.4%)	3(1.8%)	8(3.86%)	13(7.5%)	**0.03**

CPB, Cardiopulmonary Bypass; LOS, Length of Stay; LVEF, Left Ventricular Ejection Fraction; NTV, Normalized Total Variance; POD, Postoperative Day; RRT, Renal Replacement Therapy; RV, Right Ventricle

Donor undersizing (low predicted heart mass (PHM) ratio), prior sternotomy, and pre-transplant LVAD were the strongest predictors of the Low CPOI-VIS phenotype, whereas pre-hospitalization and pre-transplant IABP were associated with lower risk. Older recipient age and longer allograft ischemic time also showed weaker but significant associations (Supplemental Section 5). After balancing baseline differences, the Low phenotype remained independently associated with ECMO ≤72 hours (OR 1.07, 95% CI 1.04-1.11, *p* < 0.001), RRT (OR 1.26, 1.17-1.35, *p* < 0.001), longer ICU stay (+4.0 days, 2.8-5.2, *p* < 0.001), and higher 90-day mortality (OR 2.85, 1.06-7.70, *p* < 0.001). (Table 3).

**Table 3:** Weighted Regression Results Showing Effect of Low CPOI-VIS Phenotype on Post-transplant Outcomes (Compared to Both Moderate and High CPOI-VIS Phenotypes Combined)

	**OR (95%CI)/Median change(95%CI)**	***p* value**
Need for ECMO within 72 h	1.07(1.04-1.11)	**<0.001**
RRT	1.26(1.17-1.35)	**<0.001**
Mortality 90 day	2.85(1.06-7.7)	**<0.001**
ICU LOS (media change, days)	4.0(2.8-5.2)	**<0.001**
Mortality 1y	2.03(0.89-4.70)	0.09

LOS, Length of Stay; RRT, Renal Replacement Therapy

## Discussion

In this large, single-center cohort, we identified CPOI-VIS as a dynamic, physiology-based hemodynamic marker that accurately captures allograft performance and was strongly associated with the need for mechanical circulatory support (VA-ECMO) after heart transplantation. Both the absolute level and dynamic behavior of CPOI-VIS were strongly associated with imminent need for VA-ECMO.

Several aspects of these findings are noteworthy. First, early graft physiology is quantifiable and may potentially be predictive from the moment of reperfusion. Patients who ultimately required ECMO exhibited reduced CPOI-VIS at hour 0, with progressive divergence during the first 72 hours. This observation is consistent with prior work demonstrating that early CPOI-VIS is associated with PGD severity and adverse outcomes.^16^ The fact that the CPOI-VIS separation was evident immediately after transplantation suggests that the “physiologic imprint” of donor, recipient, and procedural stresses manifests as impaired myocardial efficiency from the outset.^17^

Second, CPOI-VIS appears to identify a reproducible physiologic range associated with increased risk of early graft dysfunction. In this cohort, internally derived thresholds clustered around 4.8 W/m², with bootstrap analyses suggesting a slightly higher range (∼7 W/m²), indicating variability in threshold estimation. Rather than representing a fixed decision boundary, this range (∼ 4-7 W/m²) may serve as a reference point to contextualize early hemodynamic deterioration. Importantly, CPOI-VIS integrates cardiac output, arterial pressure, and vasoactive support into a single physiologic measure of myocardial efficiency,^11^, ^16^ in contrast to isolated parameters such as cardiac index, MAP, or VIS, which may be misleading when interpreted independently.

Third, our exploratory analyses suggest that the clinical meaning of low CPOI-VIS depends on its trajectory rather than a single value. Although early threshold crossing was common, most patients remained ECMO-free, and a substantial proportion demonstrated recovery after the initial postoperative period. In contrast, persistent low CPOI-VIS was associated with a higher likelihood of ECMO requirement, indicating that sustained impairment carries greater clinical significance than isolated early reductions. Furthermore, among patients who crossed CPOI-VIS ≤ 4.8 W/m^2^ and required ECMO, those who underwent early ECMO had a lower observed proportion of 90-day mortality compared with those who underwent later ECMO, despite small sample size. These results align with observational data in cardiogenic shock demonstrating potential survival benefits with earlier ECMO or other mechanical support in graft dysfunction,^9^, ^10^ and reinforce the principle that timely recognition and intervention may alter the trajectory of PGD.

Fourth, low CPOI-VIS retained prognostic relevance even in the absence of ECMO. Among patients who never received ECMO, low CPOI-VIS (≤ 4.8 W/m^2^) had a significantly higher association with post-transplant mortality. This finding reinforces that low CPOI-VIS is not simply a surrogate for treatment decisions; it may reflect underlying pathophysiologic compromise that portends worse outcomes. In this sense, CPOI-VIS has the potential to help standardize PGD grading independent of variability in ECMO practice patterns across institutions. At the same time, the observation that many patients with initially low values recovered without ECMO reinforces that no single threshold is sufficient to guide intervention. Instead, CPOI-VIS should be interpreted in the context of trajectory, clinical status, and other markers of graft function. It is pertinent to mention that threshold analyses were used to anchor hypothesis-generating evaluations of physiologic deterioration and timing, rather than to define a universal decision rule. Accordingly, thresholds are likely to vary across postoperative phases, patient populations, and institutional practices, underscoring the need for prospective calibration and multicenter validation before clinical implementation.

Our observations are grounded in a well-established physiologic framework. CPOI provides a direct estimate of hydraulic power generation per body surface area, the fundamental mechanical work performed by the heart.^11^, ^15^ Fincke et al. showed that cardiac power was the strongest hemodynamic predictor of mortality in cardiogenic shock,^11^ and subsequent studies have confirmed its prognostic value in heart failure, ischemic cardiomyopathy, and valvular heart disease.^12^, ^13^, ^14^ In the transplant setting, Lim et al. linked low early CPOI to severe PGD and worse survival.¹⁶ In newly implanted hearts emerging from ischemic and reperfusion stress, a falling CPOI reflects intrinsic contractile failure, while rising VIS quantifies escalating pharmacologic compensation.^16^, ^18^ By normalizing CPOI for the intensity of vasoactive support, CPOI-VIS expresses the net efficiency of cardiac work per unit of pharmacologic drive effectively, the “effective cardiac power” of the allograft. Although Lim et al. reported that unadjusted CPOI had stronger discriminative performance than VIS-adjusted CPOI,^16^ adjusting for vasoactive support offers important methodological advantages. Both the thresholds for initiating vasoactive therapy and the specific agents and dosing strategies used vary widely across centers and clinicians. By incorporating a standardized vasoactive-inotropic score, a VIS-adjusted cardiac power index (CPOI-VIS) normalizes cardiac performance relative to the intensity of pharmacologic support. This adjustment improves comparability, generalizability, and reproducibility across institutions, making the metric less sensitive to practice-pattern variability and more suitable for multicenter implementation. Furthermore, in our analyses with a larger sample size, found excellent discriminatory performance based on CPOI-VIS.

Our machine-learning phenotyping further reinforces the biological plausibility of CPOI-VIS as a potential integrative marker of early graft vulnerability. In our cohort, the low CPOI-VIS phenotype mapped logically to established risk factors for PGD, including donor undersizing, prolonged ischemic time, LVAD explant physiology, and prior sternotomy.^16^, ^19^, ^20^, ^21^ Lower predicted heart mass ratios and older recipient age were associated with the low-efficiency phenotype, reflecting the combined impact of suboptimal size matching and reduced physiologic reserve. Prolonged ischemic time, long recognized as a driver of metabolic and reperfusion injury,^2^, ^20^ was similarly associated with lower CPOI-VIS. LVAD explant patients in this phenotype likely reflect complex biventricular interactions and chronic changes in right ventricular loading conditions that impair early graft performance.^21^ In contrast, pre-transplant IABP and in-hospital optimization (reflected as hospital admission before transplant) were associated with a lower likelihood of the low CPOI-VIS phenotype, consistent with more favorable peri-transplant hemodynamics.

Even after adjusting for these baseline characteristics and pre-transplant risk factors, low early postoperative CPOI-VIS trajectory remained independently associated with ECMO requirement, RRT, prolonged ICU stay, and higher 90-day mortality. This spectrum of complications is tightly linked to severe PGD and multi-organ dysfunction, underscoring that CPOI-VIS quantifies the same pathophysiologic process currently inferred indirectly from the need for ECMO.^2^, ^7^, ^22^

It is important to note that during the study period, CPOI and CPOI-VIS were not tracked, displayed, or used to inform clinical decision-making at our center. ECMO initiation was guided entirely by clinician judgment based on conventional hemodynamic variables, echocardiography, metabolic indices, and perceived reversibility. As a result, CPOI-VIS functioned as a latent physiologic exposure rather than a decision-contaminated metric. This design feature reduces the risk of circularity and treatment-indication bias, as the marker itself could not influence the outcome it was used to predict. In this sense, the observed associations reflect an unbiased retrospective interrogation of a physiology-based metric long established in the cardiogenic shock literature, rather than a self-fulfilling artifact of protocolized care.

In contemporary practice, assessment of early graft dysfunction already relies on integrating these multiple parameters, including cardiac index, mean arterial pressure, vasoactive support, and ventricular function, yet synthesizing these variables across time points can be complex and subject to variability in interpretation. In this context, CPOI-VIS provides a simplified, quantitative representation of global cardiac performance into a single measure of myocardial efficiency. Rather than introducing a fundamentally new signal, it offers a structured way to consolidate information that clinicians already consider. Our findings extend this concept to the post-transplant setting. We are now prospectively evaluating CPOI-VIS-guided monitoring to formally test its clinical utility, and future studies integrating CPOI-VIS trajectories with metabolic and injury markers may further clarify its relationship to myocardial injury and recovery.

### Clinical implications

By unifying perfusion output and pharmacologic drive, CPOI-VIS offers a framework for PGD grading and ECMO decision-support. Incorporating continuous or frequently updated CPOI-VIS into ICU monitoring systems could enable earlier recognition of hemodynamic decline and support timely consideration of mechanical support before multi-organ injury is established. Because the ratio normalizes for vasoactive intensity, it may also facilitate cross-center comparisons of early graft performance, reducing the subjectivity inherent in PGD definitions that rely primarily on whether ECMO is used. CPOI-VIS is amenable to integration into electronic health record dashboards and future decision-support platforms, potentially contributing to more standardized, physiology-guided transplant care. Consequently, CPOI-VIS may serve as a candidate integrative metric of graft performance that could inform future PGD definitions and care pathways. Prospective studies integrating CPOI-VIS with metabolic and injury biomarkers are needed to determine whether low CPOI-VIS reflects causal myocardial injury or reversible dysfunction.

### Limitations

Several limitations warrant careful consideration. First, this is a single-center, retrospective analysis with a relatively small number of ECMO events, which limits causal inference and generalizability. Although effect sizes were large and consistent across multiple analytic frameworks, the study is hypothesis-generating rather than causal, and the findings should not be interpreted as causative that threshold-guided ECMO initiation improves survival. Second, hour-specific rolling ROC analyses may overestimate discrimination when event counts within individual prediction windows are sparse; accordingly, we complemented these with a pooled, bootstrap-validated analysis demonstrating minimal optimism and robust overall discrimination. Threshold selection should be viewed as calibration-dependent: we used 4.8 W/m² to anchor clinically interpretable sensitivity analyses, while pooled optimization suggested a higher global cutoff (∼6.3 W/m²), underscoring the need for prospective multicenter calibration before implementation of a single universal trigger. Third, the very high discriminatory performance observed (AUC > 0.95) raises the possibility of overfitting, particularly in the setting of a rare outcome. However, the separation between groups was evident early, persisted across time, and was observable in unadjusted analyses without complex modeling, suggesting a clinically grounded phenomenon rather than purely statistical optimization. Given that CPOI directly reflects cardiac mechanical efficiency, it is biologically plausible that profound graft dysfunction would manifest as markedly reduced values. Nonetheless, external validation in independent cohorts is essential to confirm generalizability, refine the threshold, associating CPOI trajectory to metabolic profile, and define its role within prospective, protocolized decision frameworks. Finally, practice patterns, vasoactive strategies, and ECMO thresholds vary across centers, underscoring the need for multicenter prospective studies before broad implementation.

## Conclusions

In our single-center experience, CPOI-VIS was a physiologically grounded, time-dynamic metric that captures early graft performance after heart transplantation and was strongly associated with subsequent need for ECMO and adverse postoperative outcomes. By integrating cardiac output, perfusion pressure, and vasoactive support into a single measure of myocardial efficiency, CPOI-VIS provides a quantitative framework for assessing early graft dysfunction. These findings suggest that CPOI-VIS may serve as a useful marker for risk stratification and monitoring. Prospective, multicenter evaluation of CPOI-VIS as a candidate physiologic marker is required to determine its role in standardizing post-transplant monitoring, refining PGD risk stratification, and informing timely escalation of mechanical circulatory support within physiology-guided care pathways.

## Funding Statement

Mark Petrovic is supported by the NIGMS of National Institutes of Health under award number T32GM007347 and the American Heart Association under 24PRE1187121. Matthew Bacchetta is supported by Vanderbilt University Medical Center David M. Livingston Lung Transplant Memorial Fund, Cystic Fibrosis Foundation grant VUNJAK23XX0, NIH grant R01 HL171577, Vanderbilt University Medical Center Mrs. Shelley F. Kleiner and Dr. Fredric Kleiner Fund, Vanderbilt University Medical Center Ms. Dorothy Thomas Research Fund, Congressionally Directed Medical Research Programs PR212237, and DARPA N660012424024. The data set was maintained using REDCap supported under grant UL1 TR000445 from NCATS/NIH. NCATS/NIH.

## Disclosures

No disclosures to report.

## Declaration of Competing Interest

The authors declare that they have no known competing financial interests or personal relationships that could have appeared to influence the work reported in this paper.

## Footnotes

Appendix A Supplementary data associated with this article can be found in the online version at doi:10.1016/j.jhlto.2026.100597.
