# Supplementary material for: Real-time cardiac power output index predicts imminent need for extracorporeal membrane oxygenation after heart transplantation
Source: JHLT Open. 2026 May 20;13:100597. doi: 10.1016/j.jhlto.2026.100597 (PMC13265899; doi:10.1016/j.jhlto.2026.100597)
Supplement: Supplementary file 1 — Supplementary material [file mmc1.docx]

**Supplemental Materials**

Supplemental Section 1:

Trajectory description and group comparisons:

**
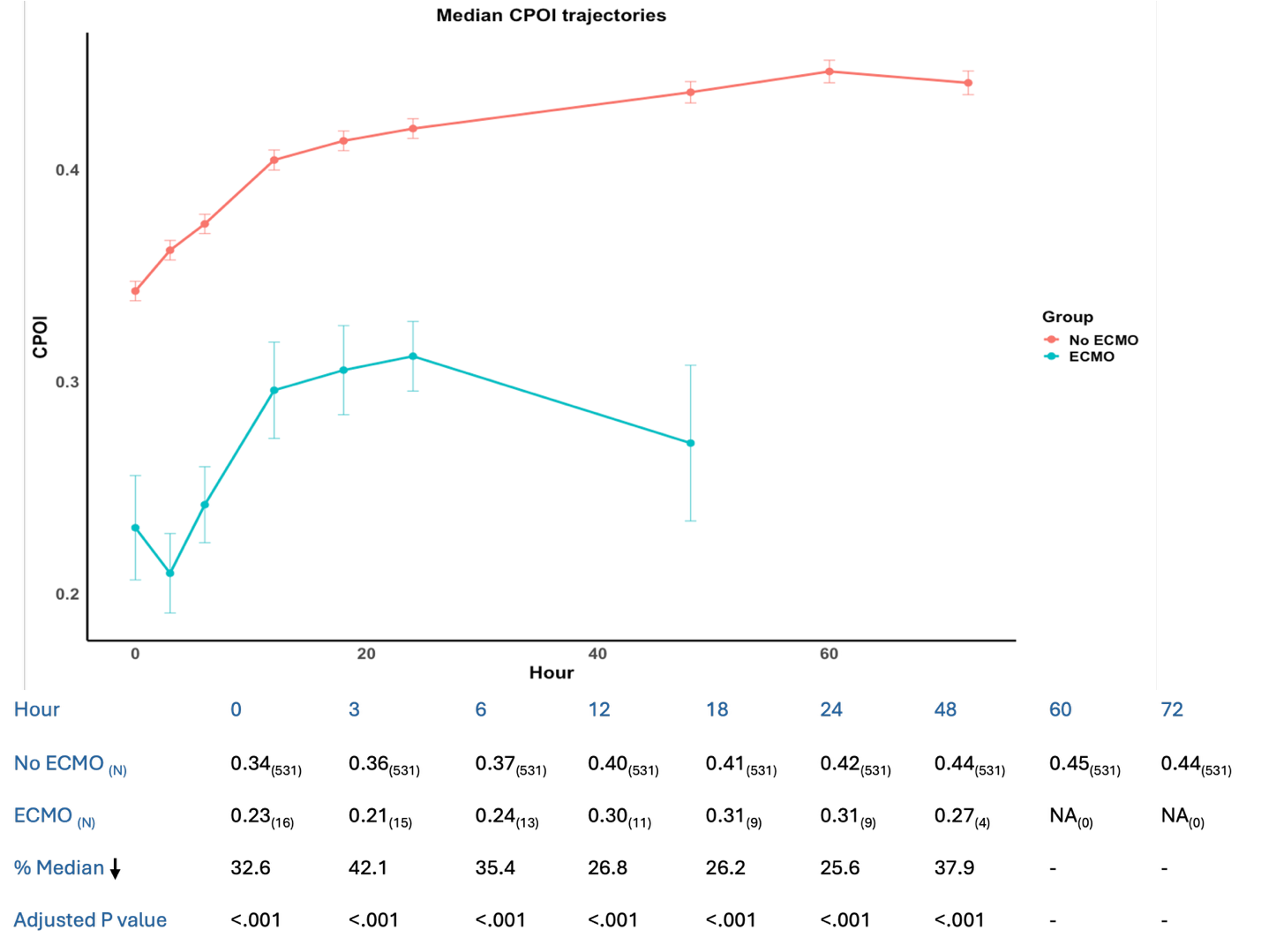
**


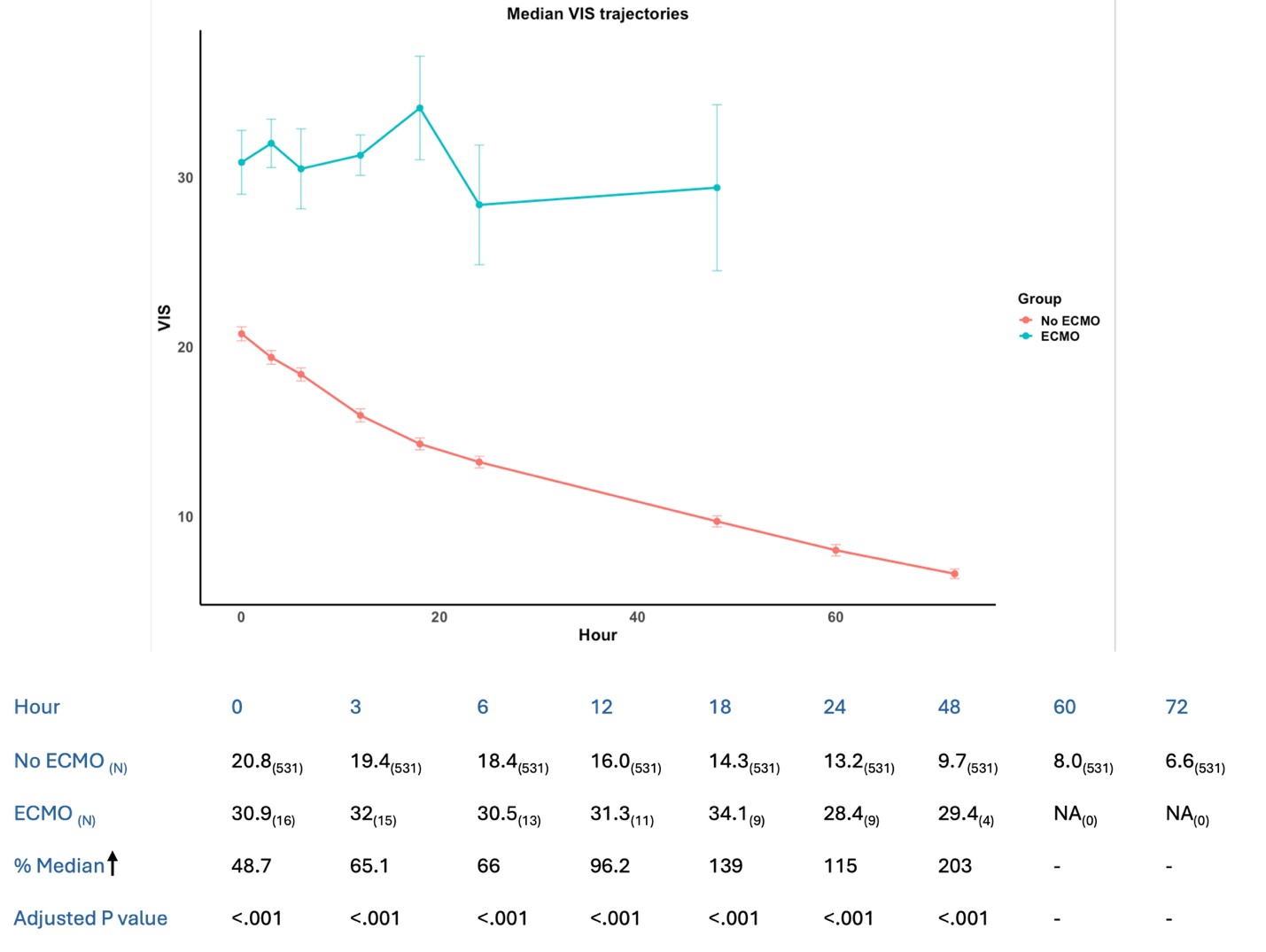


Table S1:

| **Postoperative Data** | **No Delayed ECMO**  **n = 531 (97.1%)** | **Delayed ECMO**  **n = 16 (2.9%)** | **P Value** |
| --- | --- | --- | --- |
| CPOi |  |  |  |
| Mean | 0.37 (0.31 – 0.44) | 0.25 (0.21 – 0.30) | **<0.001** |
| Median | 0.37 (0.31 – 0.44) | 0.24 (0.21 – 0.29) | **<0.001** |
| Nadir | 0.29 (0.24 – 0.36) | 0.20 (0.16 – 0.25) | **<0.001** |
| Peak | 0.47 (0.39 – 0.54) | 0.33 (0.24 – 0.42) | **<0.001** |
| Slope | 0.005 (-0.001 – 0.009) | 0.002 (-0.001 – 0.005) | 0.18 |
| VIS |  |  |  |
| Mean | 18.8 (13.9 – 24.4) | 29.0 (24.2 – 33.1) | **<0.001** |
| Median | 18.1 (13.5 – 24.0) | 29.5 (23.1 – 32.9) | **<0.001** |
| Nadir | 14.6 (10.9 – 19.8) | 24.2 (16.4 – 27.4) | **<0.001** |
| Peak | 23.7 (17.8 – 31.0) | 37.7 (34.1 – 40.8) | **<0.001** |
| Slope | -0.300 (-0.665 – 0.000) | 0.003 (-1.122 – 0.516) | 0.24 |
| CPOi adjusted by VIS |  |  |  |
| Mean | 8.71 (6.58 – 11.14) | 4.68 (3.84 – 5.65) | **<0.001** |
| Median | 8.52 (6.48 – 10.94) | 4.55 (3.90 – 5.79) | **<0.001** |
| Nadir | 6.39 (4.96 – 8.51) | 3.38 (2.88 – 4.28) | **<0.001** |
| Peak | 11.25 (8.59 – 14.35) | 6.05 (4.90 – 7.75) | **<0.001** |
| Slope | 0.18 (0.03 – 0.34) | 0.09 (-0.02 – 0.14) | **0.024** |

Supplemental Section 2:

Dynamic risk modeling risk for ECMO:

To capture the dynamic risk of ECMO, we then applied time-to-event models. Dynamic Cox models were used to estimate the instantaneous hazard of ECMO based on worsening CPOI and VIS.

In a dynamic Cox model, both CPOI and VIS were strong predictors of ECMO initiation. A 0.1-unit decrease in CPOI was associated with a >4-fold increase in instantaneous hazard of ECMO (HR 4.22, 95% CI 2.22–8.02, p <.001). Each 5-point increase in VIS was associated with a 39% higher hazard of ECMO (HR 1.39, 95% CI 1.23–1.57, p <.001) and a model C-index of 0.90.

Dynamic Landmark analyses were conducted to quantify the risk of ECMO within the next 6 hours, conditional on current CPOI and VIS values. To translate these findings into actionable bedside insights, a risk heatmap was generated from landmark analyses to show combinations of rising VIS and falling CPOI that corresponded to increased short-term ECMO risk.

In 6-hour landmark Cox models, both CPOI and VIS were strong predictors of short-term ECMO risk. Each 0.1 unit decrease in CPOI was associated with a nearly 5-fold higher hazard of ECMO within the next 6 hours (HR 4.97, 95% CI 2.46–10.0, p<0.001). Similarly, each 5-point increase in VIS increased the hazard by 40% (HR 1.40, 95% CI 1.24–1.58, p<0.001). Model discrimination was excellent (C-index 0.95).


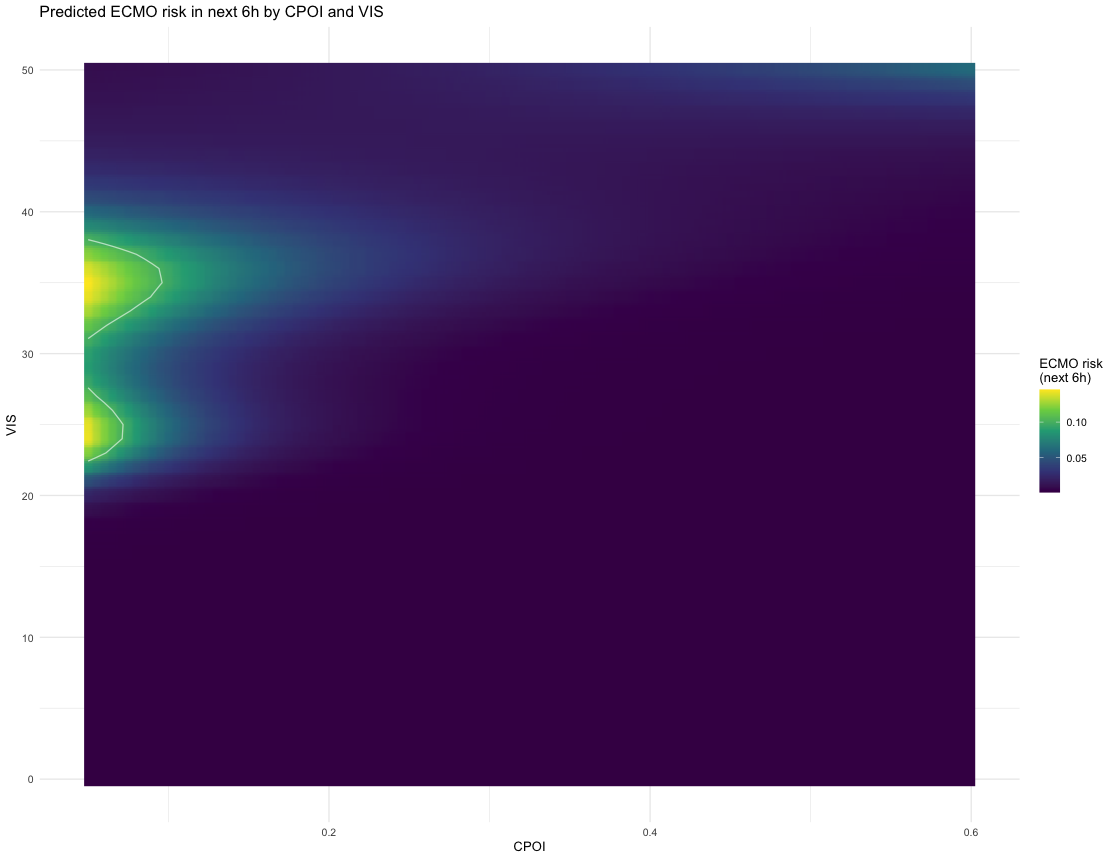


When we looked at patients’ CPOI and VIS values hour by hour, both measures were strongly linked with the chance of needing ECMO in the next 6 hours. Lower CPOI values were strongly associated with higher ECMO risk (p<0.001), while higher VIS also increased risk in a clear, nonlinear fashion (p<0.001). Importantly, we found a synergistic effect: patients with both low CPOI (< 0.25) and high VIS (> 20) were much more likely to need ECMO than those with only one abnormal value (interaction p=0.003).

In joint modeling of longitudinal CPOI and VIS trajectories with time to ECMO, the absolute level of CPOI emerged as a strong predictor of ECMO initiation, whereas the slope of CPOI over time was not informative. Specifically, lower CPOI values were strongly associated with increased instantaneous risk of ECMO (estimate –20.3, 95% credible interval –28.8 to –12.0, p<0.001). In contrast, the slope of CPOI did not show a significant association (estimate –41.0, 95% CI –482.6 to 400.8, p=0.87). Higher VIS values were independently linked with greater ECMO risk (estimate 0.071, 95% CI 0.040–0.097, p<0.001).

These findings highlight that it is the absolute thresholds of CPOI and VIS, rather than short-term fluctuations, that drive clinical risk, underscoring their role as actionable dynamic biomarkers for timely ECMO decision-making.

Supplemental Section 3:

Rolling ROC and threshold identification:

Dynamic ROC analysis was used to identify the optimal threshold of CPOI predictive of ECMO within 72 hours. A rolling ROC analysis was performed at each post-transplant hour time point to predict risk in the next 6 hours.


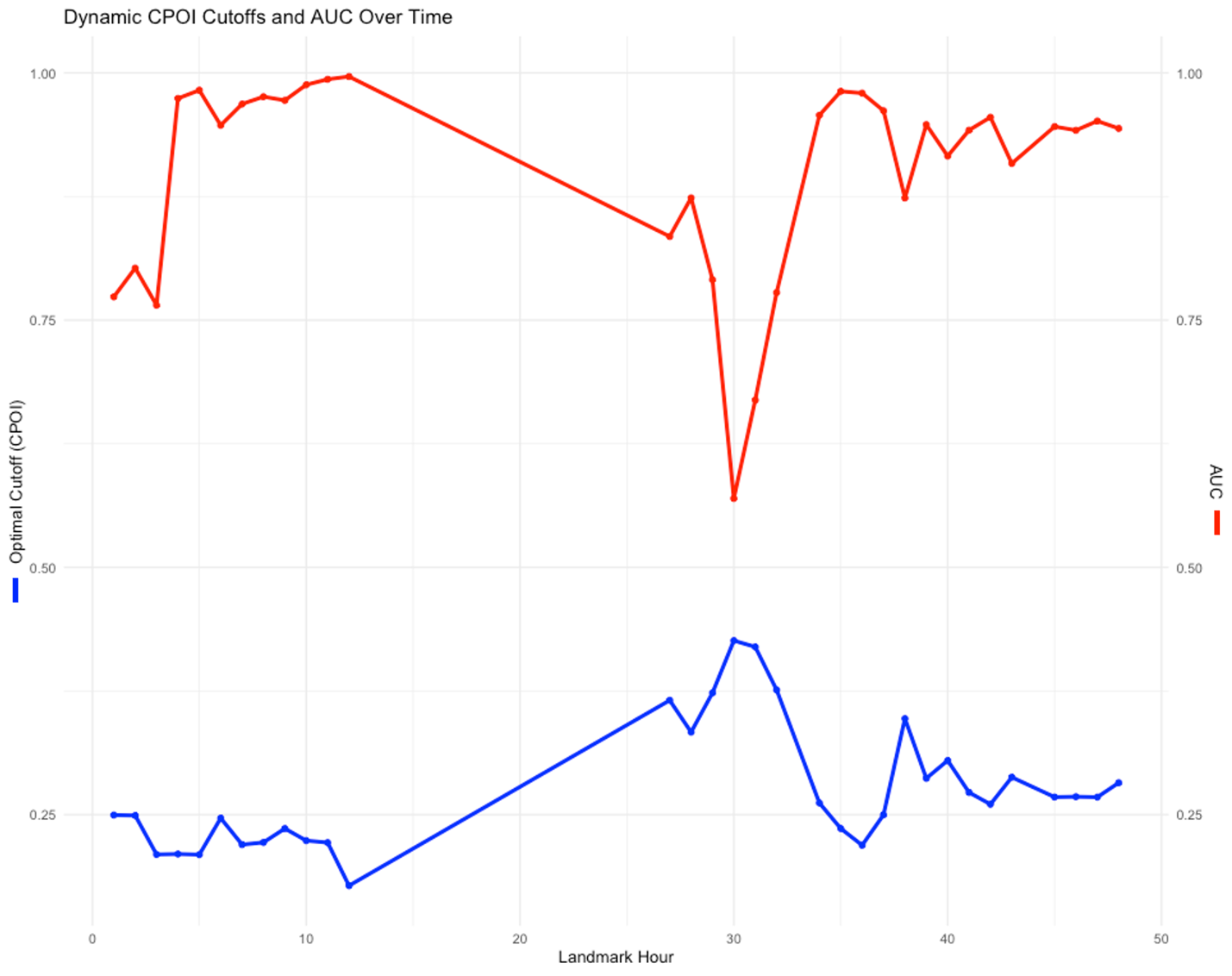


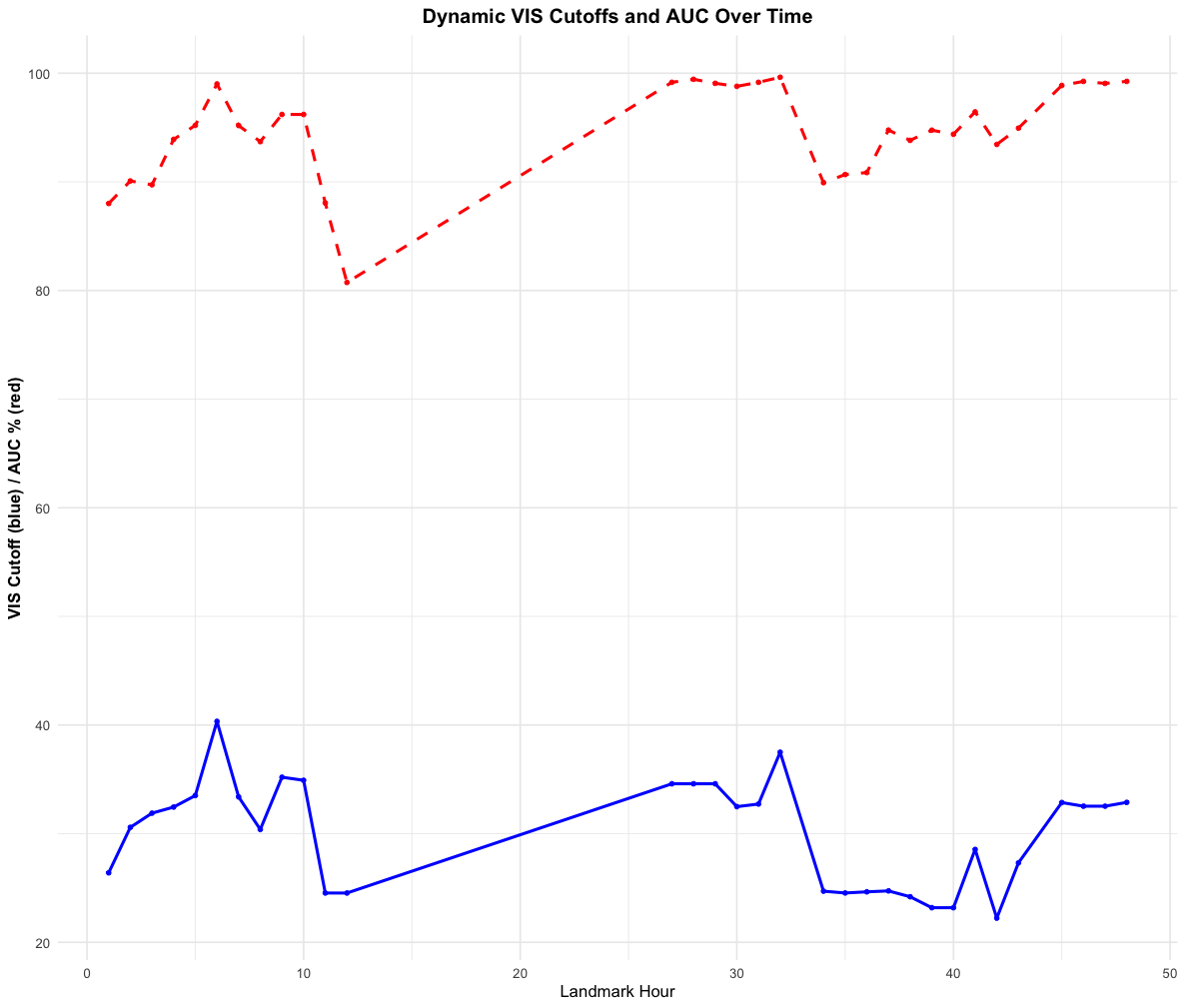
Dynamic ROC analyses demonstrated that a CPOI threshold of 0.26 (IQR 0.22–0.29) and a VIS threshold of 32 (IQR 25–33) were most predictive of ECMO initiation within the next 6 hours. Discriminatory performance was excellent for both indices, with a median AUC of 0.95 (IQR 0.86–0.97) for CPOI and 0.95 (IQR 0.93–0.99) for VIS.

A sensitivity analysis assessed whether initiating ECMO within 24 hours of crossing critical CPOI/VIS thresholds could reduce 90-day mortality compared to later initiation.

To test whether acting on these thresholds and initiate ECMO early may rescue individuals from early mortality (90day mortality), we defined a resistant state as CPOI ≤ 0.26 and VIS ≥ 32.

Supplemental Section 4:

“Early vs Late” ECMO sensitivity analysis anchored to thresholds:

For each patient, we identified the first hour that both criteria were met, then classified ECMO as early if initiated within 24 hours of that crossing and later otherwise. Among the 12 resistant patients (7 early ECMO, 5 late ECMO), there were 0 deaths at 90 days in the early group compared with 2 deaths (40%) in the late group. Using a Bayesian small-sample analysis (Jeffreys prior), the estimated 90-day mortality risk was 6.2% (95% CrI 0.007–29%) for early ECMO versus 41.7% (95% CrI 9–79%) for late ECMO. The risk difference favored early ECMO (−35.5%, 95% CrI −75% to +1.9%), with a 97% posterior probability that earlier initiation reduces mortality.


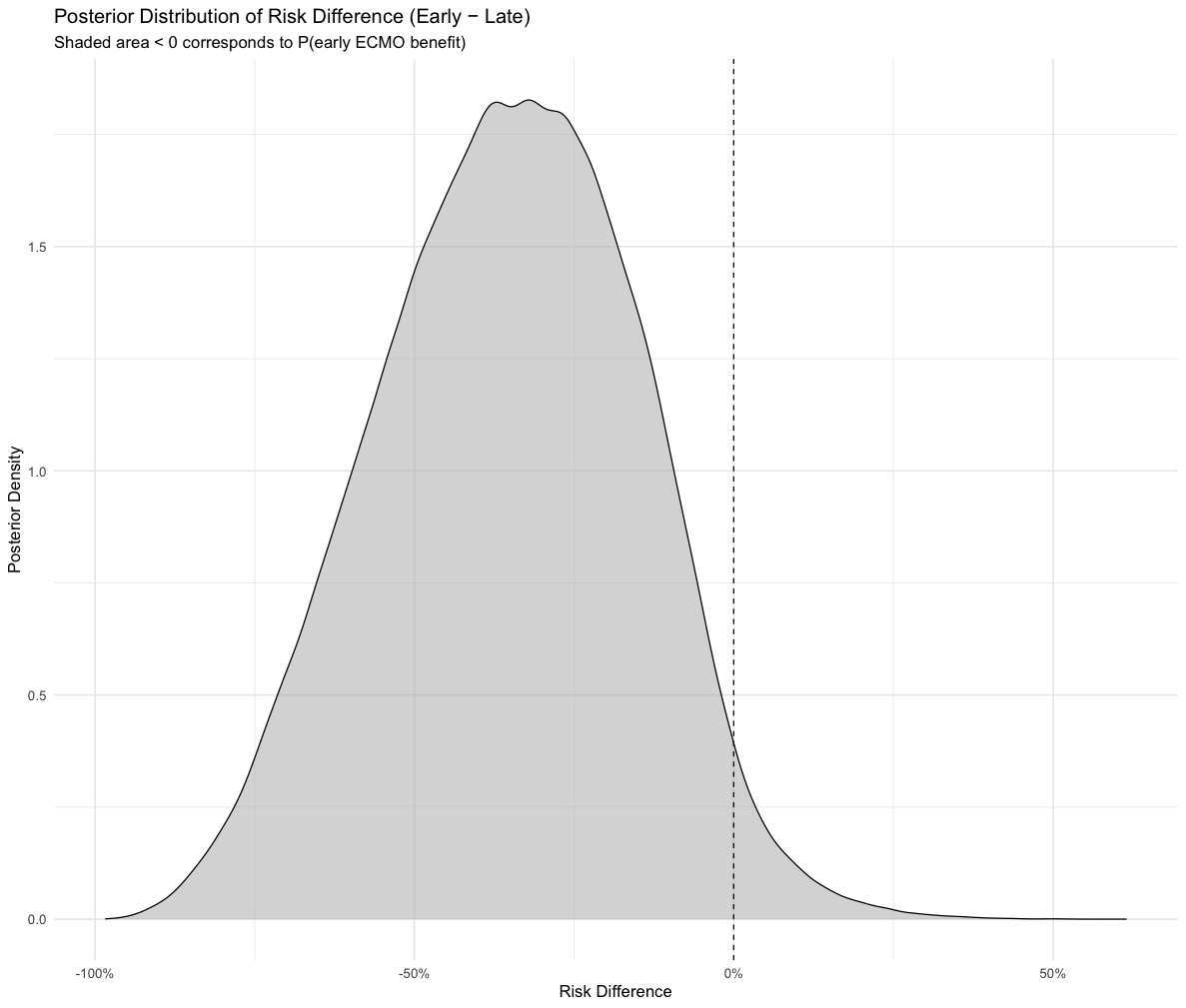


Showing reduction in the risk of mortality if ECMO was initiated within 24 hours of reaching critical CPOI and VIS.

*Mortality Risk Reduction with CPOI-VIS threshold:*


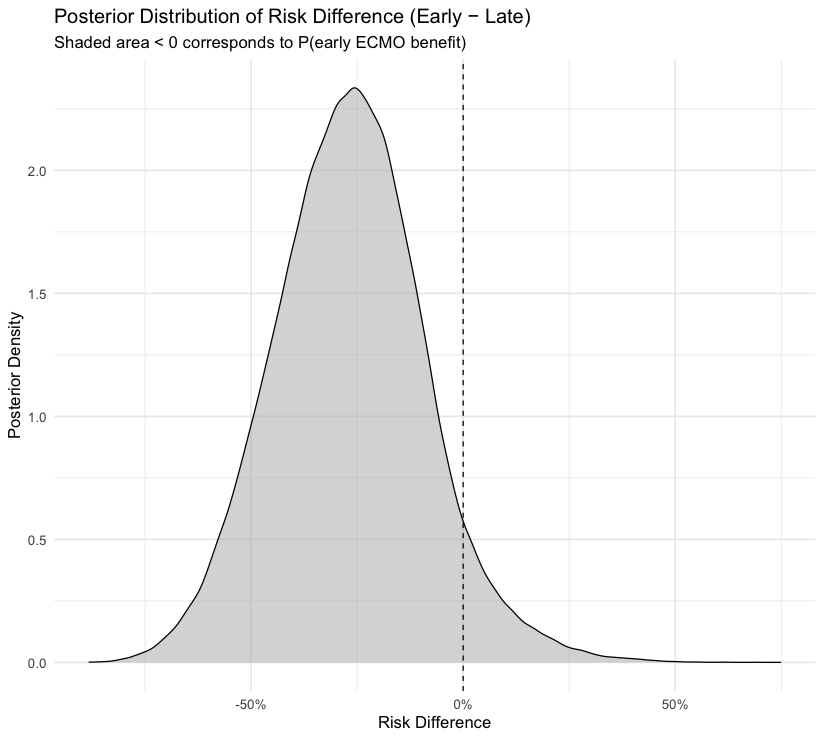


Showing reduction in the risk of mortality if ECMO was initiated within 24 hour of reaching critical VIS adjusted CPOI.

Supplemental Section 5:

Unsupervised phenotyping and independent effects on outcomes:


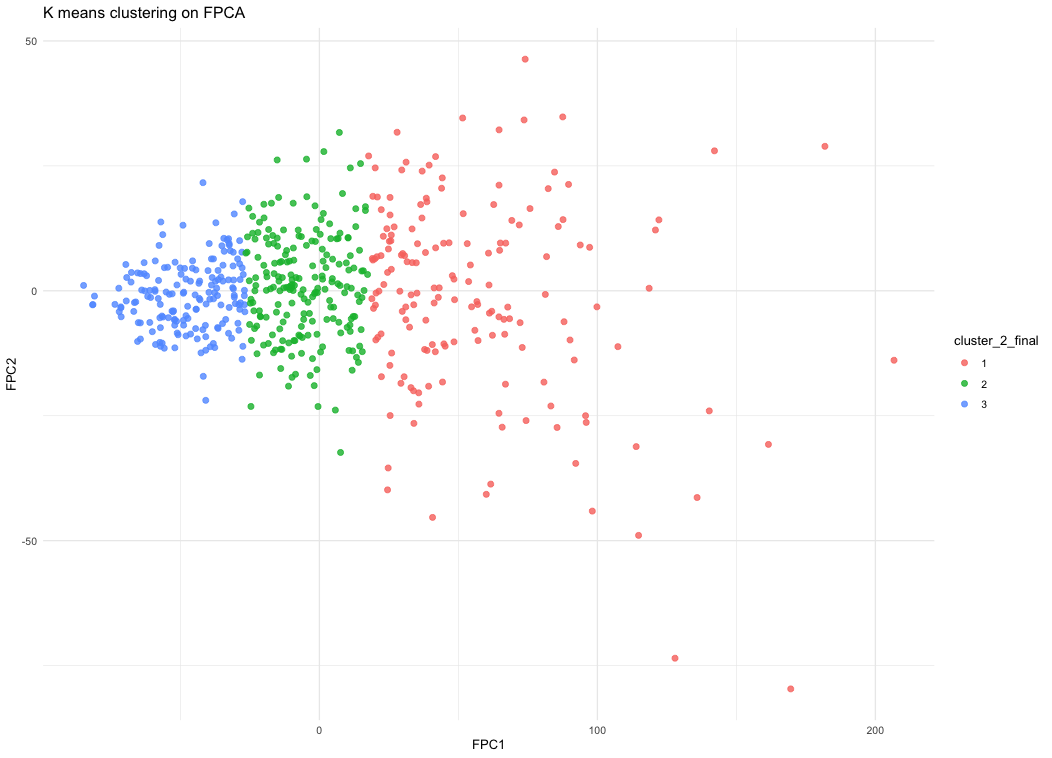


*FPC1 explained 88.7% of variance while FPCA2 explained 8.1%.*

IPTW-weighted regression model for quantifying independent effect of low CPOI-VIS on post-transplant outcomes.


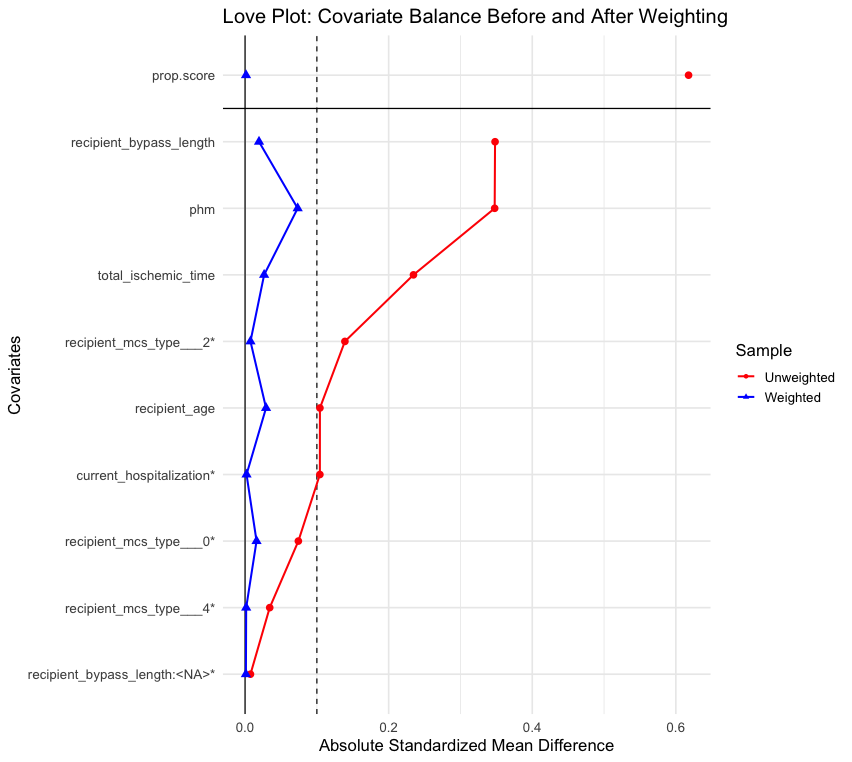


Phm: predicted heart mass ratio; total_ischemic_time: allograft ischemic time; recipient_mcs_type___2: pre-transplant LVAD; current_hospitalization: hospitalized before transplant; recipient_mcs_type___0: pre-transplant IABP; recipient_mcs_type___4: pre-transplant ECMO.

As the high VIS-CPOI group had no ECMO event, so having a three-group variable was making the model unstable (complete separation problem), so we combined high and moderately low CPOI-VIS groups and used it as a reference to quantify the risk of severely low CPOI-VIS group independently.

L1 regularized regression model:

**
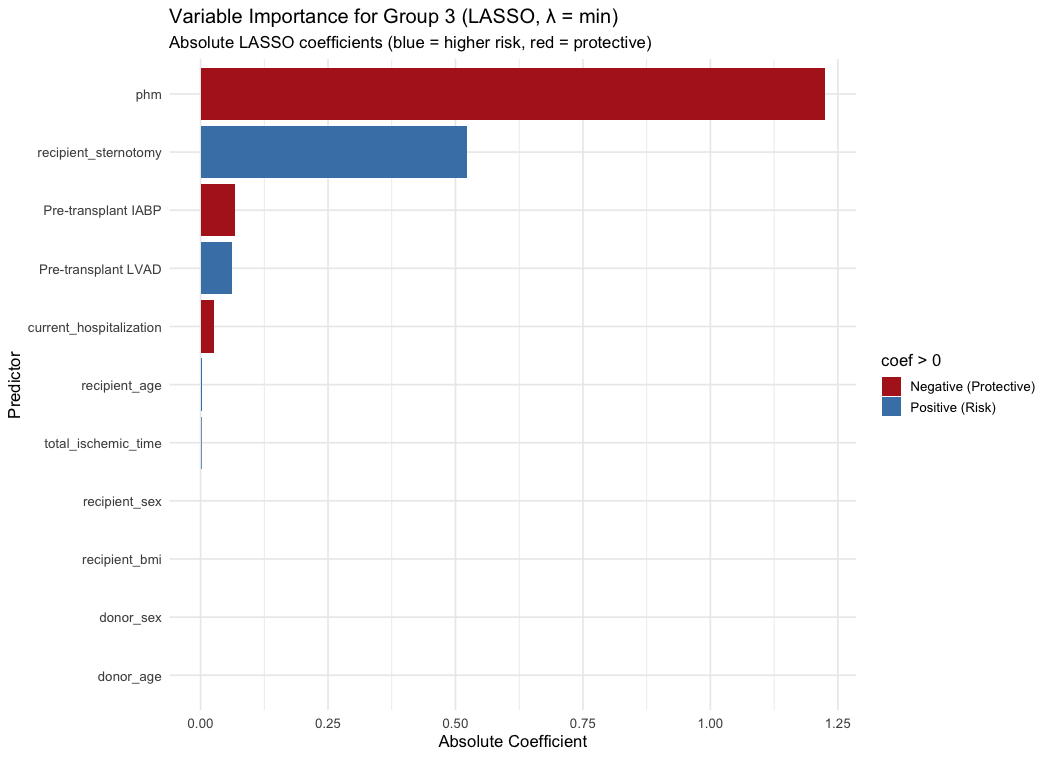
**

Phm: predicted heart mass ratio; recipient_sternotomy: prior sternotomy; IABP: Intra-aortic balloon pump; LVAD: Left Ventricular Assist Device; Current_Hospitlaization: Hospitalized before transplant; total_ischemic_time : allograft ischemic time.
